# Spontaneous Hemocholecyst in an End-Stage Renal Failure Patient on Low Molecular Weight Heparin Hemodialysis

**DOI:** 10.1155/2012/363924

**Published:** 2012-12-11

**Authors:** Konstantinos Blouhos, Konstantinos A. Boulas, Dimitrios G. Tselios, Anestis Hatzigeorgiadis

**Affiliations:** Department of General Surgery, General Hospital of Drama, End of Hippokratous Street, 66100 Drama, Greece

## Abstract

The present paper describes a case of spontaneous hemocholecyst in a patient with end-stage renal failure on low molecular weight heparin hemodialysis. The patient presented with acute right upper quadrant pain. An initial ultrasound scan demonstrated a distended gallbladder containing echogenic bile without stones. During hospitalization the patient became febrile, and jaundiced, developed leukocytosis, and had an elevation in serum bilirubin, transaminases, and alkaline phosphatase. A new ultrasound demonstrated a thick-walled gallbladder containing echogenic bile and pericholecystic fluid. MRI depicted a distended gallbladder containing material of mixed signal intensity and a normal biliary tract. Open cholecystectomy revealed a gallbladder filled with blood and clots, and transcystic common bile duct exploration flushed blood clots out of the bile duct. To our knowledge this is the second case of spontaneous hemocholecyst reported in the literature as a consequence of uremic bleeding and LMWH hemodialysis in the absence of other pathology.

## 1. Introduction

Uremic bleeding is a well-recognized complication in patients with renal failure. The clinical importance of bleeding associated with chronic renal failure (CRF) itself is, however, difficult to assess, especially as various dialysis techniques, comorbidities, and medications are known to affect platelet aggregation and/or the coagulation cascade [1]. Although renal dialysis patients are at an increased risk for developing acute gastrointestinal bleeding, bleeding confined to the gallbladder (GB) is extremely rare. This study presents a case of hemocholecyst in a patient with end-stage renal failure on low molecular weight heparin (LMWH) hemodialysis, which is an exceptionally rare event, especially when there is no concurrent pathology as in our patient case.

## 2. Case Presentation

**Figure 1:**
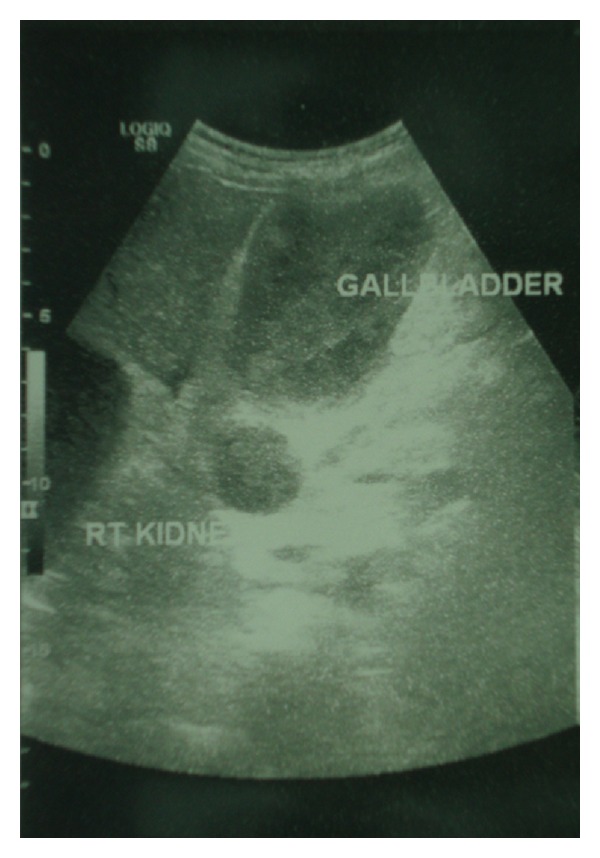
Ultrasound scan demonstrating a distended gallbladder containing echogenic bile (thick sludge or pus or blood-like) without stones and a simple right kidney cyst.

A 70-year-old male with end-stage renal failure was referred to our surgical department owing to abdominal pain localized in the right upper quadrant, 2 hours after hemodialysis. The patient suffered from CRF secondary to mesangioproliferative glomerulonephritis and commenced short dialysis sessions using routinely enoxaparin (40 mg) via the arterial port 3 to 4 minutes prior dialysis, as anticoagulation. Direct questioning revealed no other past medical history and no use of oral anticoagulants or antiplatelet medications. Physical examination revealed localized tenderness and palpable mass in the right upper quadrant. The patient was hemodynamically stable. Axillary temperature was 36.6°C. Initial laboratory and coagulation tests were normal. An ultrasound scan demonstrated a distended GB containing echogenic bile without stones in the lumen and no enlargement of the biliary (intra- and extrahepatic) ducts or hepatic or pancreatic focal lesions (Figure 1). The exact nature of this material was thought to represent thick sludge, pus, or blood. 

**Figure 2:**
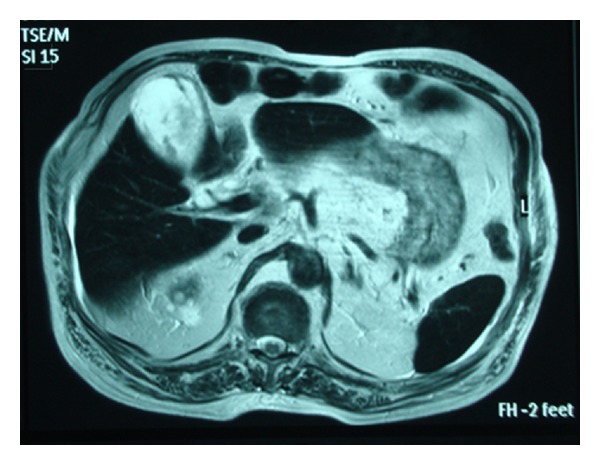
T2-weighted MRI showing a distended gallbladder enhanced by mixed signal intensity (hemorrhagic or exudative-like) component.

**Figure 3:**
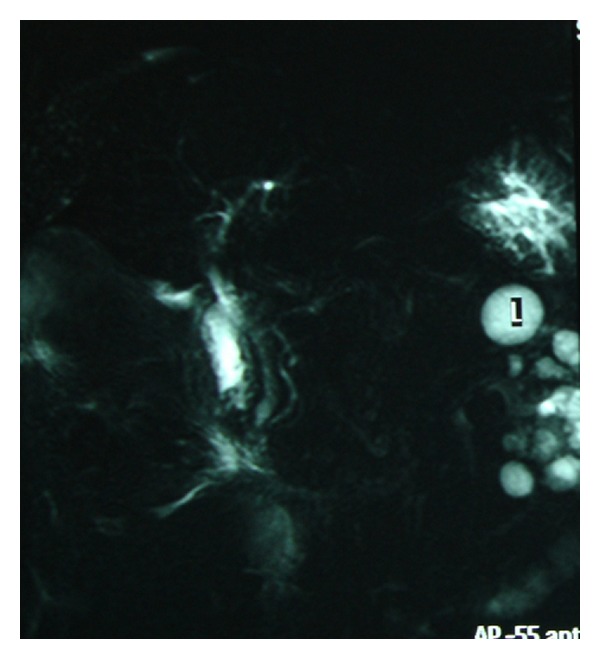
MRCP demonstrating normal appearance of the biliary tree.

On the second day of hospitalization, the patient became febrile and developed right upper guardant guarding. Mild jaundice was also present. The patient remained hemodynamically stable. A complete blood count revealed white blood cell count (WBC) 16840/mm^3^, absolute neutrophils count 14730/mm^3^, and no differences in hemoglobin. Biochemical tests were remarkable for hyperbilirubinemia and elevation in transaminases, alkaline phosphatase (ALP), and gamma-glutamyl transpeptidase (GGT). A new ultrasound scan obtained and showed evidence of a distended thick-walled GB containing echogenic bile, pericholecystic fluid and a biliary tract with normal echoes and without dilation. Heavily T2-weighted MRI depicted a distended wide double borders (cholecystitis-like) GB enhanced by mixed signal intensity (hemorrhagic or exudative-like) component and intraluminal air (Figure 2). In MRCP, biliary tract appeared normal (Figure 3). 

**Figure 4:**
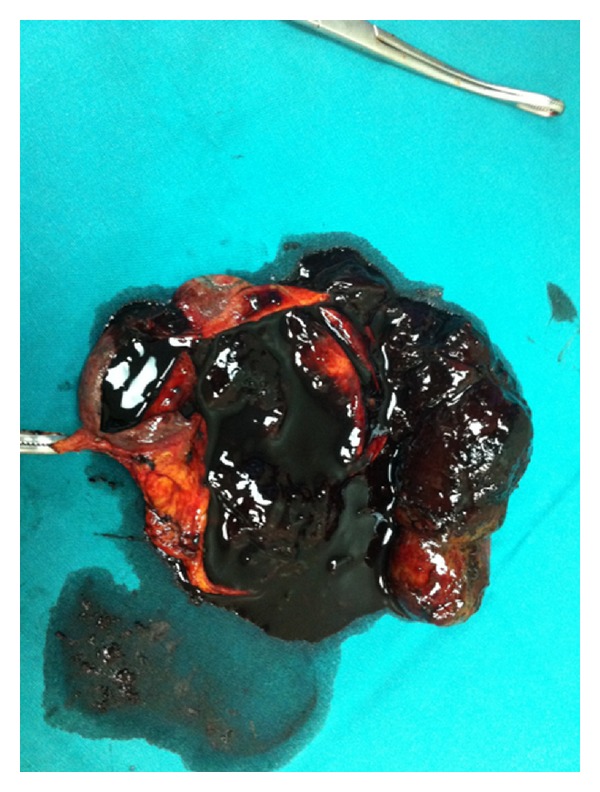
Cut operative specimen showing a mixed material of blood, clots, and bile creating a mold of the gallbladder lumen.

On the third day of hospitalization, the patient underwent laparotomy. Intraoperative evacuation of the distended GB showed evidence of blood and clots. Open cholecystectomy performed (Figure 4) combined with transcystic common bile duct exploration (TCBDE) which flushed blood clots out of the common bile duct (CBD). During the next 2 postoperative days, the patient experienced some blood loss in the abdominal drain tube and hematic stools (melena), but no transfusion was required. WBC count, liver enzymes, serum bilirubin, ALP and GGT gradually decreased during the following 5 days and the patient was then sent home.

## 3. Discussion

Blood in the biliary tract may arise from liver, extrahepatic bile ducts, GB, and pancreas. The term hemocholecyst (HC) refers to bleeding confined to the GB [2]. HC can be classified according to etiology, as primary (spontaneous) or secondary (Table 1). In our patient case, HC occurred spontaneously due to uremic bleeding diathesis and LMWH dialysis, as there was no history of trauma or preexisting gallbladder pathology.

HC disease can be considered as a spectrum of clinical entities which includes simple or complicated HC. Complications include (a) acute cholecystitis, (b) hemobilia (HB) with or without cholangitis, (c) pancreatitis, and (d) acute upper gastrointestinal hemorrhage. The sonographic appearance of blood clots within the GB appears as clumps of nonshadowing, nonlayered echogenic material and should be differentiated from other causes of echogenic bile seen with pus or thick sludge. CT scan can show homogeneous or inhomogeneous high density material and occasionally can depict active extravasation of intravenous contrast material inside the GB lumen. MRI can reveal a GB containing material of mixed signal intensity consistent with blood products [3]. It is difficult to provide an algorithm for management of HC disease. The presence of migrating blood clots into the CBD should be evaluated before decision making of treatment. Simple HC can be managed conservatively. In case of complications, treatment of choice is laparoscopic cholecystectomy combined with laparoscopic TCBDE in an attempt to flush migrating clots out of the CBD. Laparoscopic choledochotomy can be performed if TCBDE fails or is contradicted (friable cystic duct, intrahepatic clots, multiple large clots) [4]. In our case, the diagnosis of HC was not prompt. The GB content was thought to represent thick sludge, pus, or blood. Differential diagnosis included GB empyema, emphysematous cholecystitis, or complicated HC. Although, early laparoscopic cholecystectomy is a safe approach in the management of acute cholecystitis in chronic hemodialysis patients [5], we chose open cholecystectomy because of the high risk of conversion in patients with an increased risk of GB gangrene, as our patient [6].

Disturbances in hemostasis are common complications of kidney diseases. Both bleeding diathesis and thromboembolism have been identified. The pathogenesis of uremic bleeding is multifactorial. It has been attributed to platelet dysfunction, abnormal platelet-vessel wall interactions, and altered rheological properties of the blood flow. However, the clinical importance of bleeding associated with CRF is difficult to assess as various dialysis techniques are known to affect the coagulation cascade (such in our patient case). It is well recognized that anticoagulation is required during hemodialysis to maintain patency of the extracorporeal circuit. Traditionally, unfractionated heparin (UFH) has been used in patients with end-stage renal failure. In Western Europe, LMWHs are now widely used for anticoagulation during hemodialysis. In our institution, patients with end-stage renal failure and without bleeding diathesis or heparin-induced thrombocytopenia are dialyzed using enoxaparin (40 mg) via the arterial port 3 to 4 minutes before commencing short dialysis sessions. Purported benefits of LMWHs are reduced dialyzer clotting and reduced cost and no requirement for routine clinical monitoring. However, duration of the anticoagulation effect is prolonged following a bolus injection of LMWH enoxaparin with anti-Xa activity levels of 0.4 IU/mL 10 hours postdialysis and with a persistent elevation of >0.1 IU/mL at 24 hours [7].

In conclusion, HC represents a rare but serious complication in end-stage renal failure patients. To our knowledge spontaneous HC has only been reported once in the literature as a consequence of uremic bleeding and LMWH hemodialysis in the absence of other pathology [8]. Despite the small number of cases documented, physicians should keep this complication in mind when facing unusual symptoms in hemodialysis patients.

## Figures and Tables

**Table 1:** Causes of hemocholecyst reported in PubMed using hemocholecyst, hemobilia, and hemorrhagic cholecystitis as search terms.

Primary hemocholecyst		Bleeding diathesis	
		Renal disease	
		Critical illness	
Secondary hemocholecyst	Intraluminal	Gallstone, parasites	
	Intramural	Inflammatory process	Cholecystitis
		Neoplasms	
		Primary	GB cancer, polyps, hemangiomas, heterotopic gastric tissue
		Secondary	Gastric, hepatic, and pancreatic cancer, melanoma
		Traumatic	Iatrogenic, accidental
		Vascular	HSP, SLE
			GB varices
	Extrinsic	Duodenal ulcer	
		Aneurysmal disease	
		Idiopathic	
		Abdominal trauma	
		Inflammatory	Cholecystitis, hepatic abscess
		Arteritis	PAN, HPS, SLE,
		Connective tissue defect	MCTD, Marfan syndrome

*HSP: Henoch-Schönlein purpura, SLE: systemic lupus erythematosus, GB: gallbladder, PAN: polyarteritis nodosa, and MCTD: mixed connective tissue disease.
